# An Unusual Case of Breast Implant-Associated Anaplastic Large Cell Lymphoma

**DOI:** 10.1155/2022/4700787

**Published:** 2022-06-09

**Authors:** Sarah Premji, Andreia Barbieri, Christine Roth, Eric M. Rohren, Gustavo Rivero, Sravanti P Teegavarapu

**Affiliations:** ^1^Department of Medicine, Baylor College of Medicine, Houston 77030, TX, USA; ^2^Department of Pathology and Immunology, Baylor College of Medicine, Baylor St. Luke's Medical Center, Houston 77030, TX, USA; ^3^Department of Radiology, Baylor College of Medicine, Houston 77030, TX, USA; ^4^Department of Medicine, Section of Hematology-Oncology, Baylor College of Medicine, Houston 77030, TX, USA; ^5^Dan L Duncan Comprehensive Cancer Center, Baylor College of Medicine, Houston, TX 77030, USA

## Abstract

**Introduction:**

Breast implant-associated anaplastic large cell lymphoma (BIA-ALCL) is a rare disease entity associated with textured breast implants. Though the clinical course is typically indolent, BIA-ALCL can occasionally invade through the capsule into the breast parenchyma with spread to the regional lymph nodes and beyond including chest wall invasive disease.

**Case:**

We present the case of a 51-year-old female with a history of bilateral silicone breast implants placed approximately twenty years ago who presented with two months of progressively enlarging right breast mass. Ultrasound-guided biopsy of right breast mass and right axillary lymph node showed CD 30-positive *ALK*-negative anaplastic large cell lymphoma, and staging work up showed extension of the tumor to chest wall and ribs consistent with advanced disease. She received CHP-BV (cyclophosphamide, doxorubicin, prednisone, and brentuximab vedotin) for six cycles with complete metabolic response. This was followed by extensive surgical extirpation and reconstruction, radiation for residual disease and consolidation with autologous stem cell transplant. She is currently on maintenance brentuximab vedotin with no evidence of active disease post autologous stem cell transplant.

**Conclusion:**

Treatment guidelines for advanced chest wall invasive BIA-ALCL are not well defined. Lack of predictive factors warrants mutation analysis and genetic sequencing to identify those at highest risk of progression to chest wall invasive disease. This rare case highlights the need for definitive consensus on the optimal management of chest wall invasive BIA-ALCL.

## 1. Introduction

Breast implant-associated anaplastic large cell lymphoma (BIA-ALCL), an ALK-negative ALCL associated with textured breast implants, was first recognized in 1997 [1]. The lifetime prevalence of BIA-ALCL is 33 per 1 million persons with textured breast implants [2]. Most patients have excellent outcomes following surgical resection alone. However, in rare cases, BIA-ALCL invades through the capsule and spreads beyond the breast parenchyma to regional lymph nodes requiring systemic chemotherapy [3]. Here, we report a case of aggressive chest wall invasive BIA-ALCL which was successfully treated with a combination of surgery, chemotherapy, radiation, and stem cell transplant. The ideal treatment approach to chest wall invasive BIA-ALCL remains unclear, and this case highlights the need for consensus to optimally manage chest wall invasive BIA-ALCL.

## 2. Case Presentation

**Figure 1:**
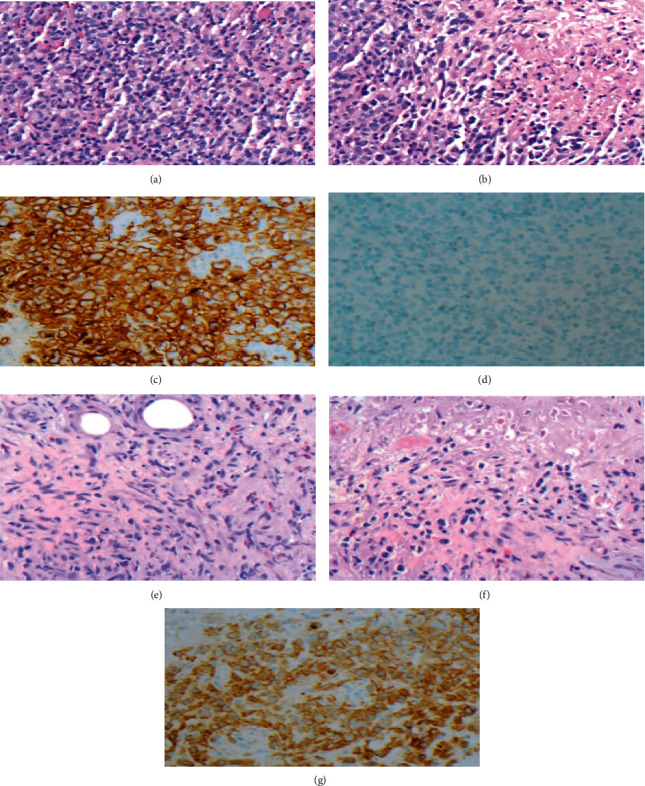
Right axillary lymph node biopsy. (a) Proliferation of large, atypical cells with irregular nuclear contours, vesicular chromatin, and moderately abundant eosinophilic cytoplasm, with background small lymphocytes and eosinophils. (b) Proliferation of large, atypical cells alternating with areas of necrosis. (c) Lymphoma cells stain positive for CD30. (d) Lymphoma cells stain negative for ALK. Right chest wall mass biopsy with (e) large lymphoma cells within background fibrosis, small lymphocytes, and eosinophils (f) with associated adjacent necrosis (g) with lymphoma cells staining positive for CD30.

**Figure 2:**
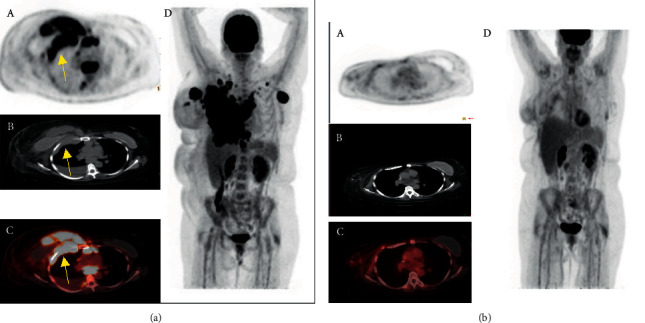
(a) Baseline: (A) axial PET, (B) CT, and (C) fused images from an FDG-PET/CT scan performed at baseline demonstrate a large, infiltrative mass in the right chest wall measuring approximately 13 x 10 x 20 cm. The mass spans the subcutaneous, muscular, and chest wall compartments with extension into the pleural/extrapleural compartments (arrows). (D) A maximum intensity projection (MIP) image shows the large extent of disease. (b) Post treatment: (A) Axial PET after treatment with 6 cycles of CHP/BV, Surgery and XRT, (B) CT, and (C) fused images with stable postsurgical changes showing (d) overall significant positive response with slight interval increase in interstitial opacity in the central right breast with low-grade FDG uptake thought to be related to inflammation post radiation.

A 51-year-old female with history of bilateral retropectoral silicone breast implants placed in 1996 presented with a two-month history of progressively enlarging right breast. Imaging including ultrasound and MRI showed a large infiltrative mass involving the right medial chest wall musculature with right axillary adenopathy along with a peri-implant fluid collection. Ultrasound guided biopsy of right chest wall mass in November 2019 showed a CD30-positive, *ALK-*negative ALCL which was negative for *DUSP22* and *TP63* gene rearrangements (Figure 1). Staging PET-CT showed a dominant, coalescent, and infiltrative 13 × 10 × 20 centimeter mass centered in the right anterior chest wall with extension into the pleural/extrapleural compartment along with involvement of regional lymph nodes and osseous invasion of the ribs consistent with advanced disease (Figure 2(a)). She was started on neoadjuvant chemotherapy with CHP-BV (cyclophosphamide, doxorubicin, prednisone, and brentuximab vedotin) and completed 6 cycles in May 2020 achieving a complete metabolic response. In July 2020, she underwent an anterior thoracotomy with resection of the right chest wall tumor, as well as ribs 3–5, partial sternectomy, en bloc removal of the right breast implant, right chest wall reconstruction with mesh/bone cement, thymectomy, serratus advancement flap, and adjacent tissue transfer. Her postoperative course was complicated by chest wall incision necrosis requiring debridement, washout and tissue transfer. Pathologic evaluation revealed viable residual tumor in the sternum, thymus, right anterior chest wall and lymph node samples. She went on to receive 36 Gy of radiation in 1.8 Gy fractions which she completed in November 2020. Restaging PET showed a complete metabolic response with SUVmax 3.9 and a Deauville score of 3 (Figure 2(b)). This was followed by consolidative auto-SCT in February 2021 with abbreviated BEAM where she received 50% of the conditioning regimen (carmustine 300 mg/m2 × 1 day, etoposide 200 mg/m2 × 2 days, cytarabine 200 mg/m2 × 2 days, and no melphalan) due to compliance issues. She engrafted well with no complications post auto-SCT. Three months post auto-SCT, she was started on maintenance BV which was stopped after 12 cycles due to worsening neuropathy. She is currently doing well about a year post auto-SCT with no evidence of active disease.

## 3. Discussion

ALCL is a rare *T*-cell lymphoma accounting for only 6% of all breast lymphomas [4]. The pathogenesis of BIA-ALCL is not well defined. It has been postulated *T*-cell activation due to sustained chronic inﬂammation mediated by a higher bacterial load associated with highly textured implants may be associated with development of *T*-cell malignancies [5]; however, no specific bacterial species have been found to predispose to BIA-ALCL [6]. In contrast to other ALCLs, BIA-ALCL are typically triple negative, negative for underlying *ALK, DUSP 22,* and *TP63* mutations. Activating mutations such as *JAK1* and *STAT3* have been implicated in excess expression of *T*-cell associated cytokines, notably, IL-6, TGF-*β*, and IL-10 along with activation of the JAK/STAT signaling pathways leading to accelerated division of lymphocytes predisposing to malignant changes. Further studies are being performed to determine if biomarkers such as SATB1 and JunB could be precursors to development of BIA-ALCL in an effort to identify these patients earlier to initiate treatment [7]. The median time to development of BIA-ALCL is 9 years with a wide range from 2 to 32 years [8]. Although the most common presentation is a peri-implant effusion, 30% of patients present with a palpable breast mass and up to 20% develop lymphadenopathy. 83% of BIA-ALCL patients are diagnosed at stage I and only 7% present with stage IV disease. The majority of patients with BIA-ALCL presenting with early-stage disease are known to have excellent prognosis. Complete capsulectomy along with removal of implant and all evidence of disease has shown improved event free and overall survival in BIA-ALCL [9]. On the other hand, those presenting with a mass tend to have lower survival and increased risk of death. In patients with advanced disease, NCCN guidelines recommend using a combination of chemotherapy (anthracycline-based chemotherapy regimens such as CHOP or CHOEP)^+/−^ radiation. BV in combination with frontline chemotherapy has shown complete response rate of 92% with overall response rate of 100% [10] in CD30-positive peripheral *T*-cell lymphomas. In fact, BV in combination with systemic chemotherapy was approved for frontline treatment of systemic ALCL and CD30 positive peripheral *T*-cell lymphomas following an overall survival benefit when compared to chemotherapy alone [11]. Case reports of BV as monotherapy has also shown durable response in both limited and advanced stage BIA-ALCL in those unable to receive anthracycline based therapy. [12, 13].

However, the optimal management approach to advanced disease remains yet to be determined. There are no prospective trials to guide the management of patients with disseminated disease. Most therapies have been extrapolated from the treatment experience of primary cutaneous and systemic ALCL. In a recent review, 39 patients diagnosed with advanced BIA-ALCL showed higher frequency of limited surgery, chemotherapy, salvage chemotherapy, external beam radiation therapy, and autologous stem cell transplantation when compared with a control group of early-stage BIA-ALCL. The rate of definitive surgery was lower as well as the time to surgery prolonged in advanced disease [14]. While delay in surgery is associated with inferior outcomes, the precise timing of surgery is unclear especially in those with aggressive chest wall invasive disease who may require neoadjuvant chemotherapy to help achieve improved surgical margins and better response rates. Coombs et al. [15] reported two such aggressive chest wall invasive BIA-ALCL cases who were successfully treated with a combination of neoadjuvant chemotherapy followed by chest wall resection and composite reconstruction for residual disease after systemic treatment. This data suggests that chest wall invasive disease may warrant a multimodality approach for improved response rate including use of neoadjuvant chemotherapy prior to definitive surgery. In fact, chest wall infiltration is a critical prognostic factor in BIA-ALCL influencing the possibility of performing a surgical radical tumor extirpation [16]. These uncommon neoplasms also represent a clinical challenge for surgeons as incorrect diagnosis, incomplete resection and unsuccessful reconstruction of thoracic wall defects have resulted in high rates of perioperative morbidity and mortality [17]. Globally, this highlights the need for definitive consensus regarding surgery and systemic therapy. Radiation for BIA-ALCL is mostly recommended for incomplete resection or residual disease though long-term toxicity to heart and lungs is unknown. Data on stem cell transplant in BIA-ALCL are limited. Collins et al. [14] reported that 8/39 (20.5%) advanced BIA-ALCL patients underwent an auto-SCT. Most of them had relapsed or refractory cases. 7/8 (87.5%) who underwent auto-SCT remain in CR, the longest follow-up to date being 120 months.

There are some limitations in our case report. Firstly, the factors contributing to extensive chest wall involvement in our patient are unknown. It would be interesting to coalesce larger number of advanced disease cases to determine whether lactate dehydrogenase (LDH) and other potential biomarkers such as beta-2-microglobulin (B2M) retain predictably for chest wall expansion. Secondly, the lack of genomic sequencing restricts our ability to correlate our patient presentation with aggressive genomic features. Despite this, our case demonstrates remarkable benefit of neoadjuvant chemotherapy prior to surgery, upfront consolidation with stem cell transplant followed by CD30 monoclonal antibody maintenance for advanced chest wall invasive BIA-ALCL. This observation is particularly of interest given the known hyper-progression phenomena previously associated with rapid chest wall involvement in the context of indolent disease when partially excised although that was not our patient's case. In near future, investigation of disease predictors and mutational signature for aggressive BIA-ALCL could assist in identification of hyper-progression cases. Most of screening continues to rely on routine mammogram in asymptomatic patients. Additionally, there is no known intervention to reduce the risk of aggressive disease. It is important to detect early seroma development to perform ultrasound/MRIs to guide aspiration of effusion. *T*-cell hyperplasia linked with bacterial biofilms is associated with BIA-ALCL suggesting that reducing infection risk, early detection of capsular contraction can reduce the activation of lymphocytes and possible conversion to BIA-ALCL [18]. Our case highlights the need to determine the optimal timing for surgical intervention as well as consensus regarding treatment paradigm for this uncommon group of patients with aggressive chest wall invasive BIA-ALCL. To date, several unresolved questions remain, which include as follows: (1) would aggressive chest wall invasive cases benefit from consolidation with auto-SCT upfront? (2) Is there a role for maintenance therapy in BIA-ALCL? Finally, our case reinforces the concept that maintenance BV after auto-SCT may improve the outcome of patients diagnosed with *T*-cell lymphomas [19] and should be considered in aggressive chest wall invasive disease to reduce recurrence. Ultimately, cooperative large-scale studies may assist in determining best therapy for aggressive chest wall invasive BIA-ALCL.

## 4. Conclusion

The treatment paradigm for chest wall invasive BIA-ALCL is not well delineated. Our case here supports the use of a multimodality approach including use of neoadjuvant chemotherapy, surgery, radiation, consolidation with stem cell transplant, and maintenance therapy post transplant for this aggressive variety of BIA-ALCL. Further research looking into the predictors of aggressive disease is much needed. Future studies with randomized control trials will help develop an evidence-based algorithm for this rare type of BIA-ALCL.

## Data Availability

No data were used to support this study.
